# Large-Scale Trends in the Evolution of Gene Structures within 11 Animal Genomes

**DOI:** 10.1371/journal.pcbi.0020015

**Published:** 2006-03-03

**Authors:** Mark Yandell, Chris J Mungall, Chris Smith, Simon Prochnik, Joshua Kaminker, George Hartzell, Suzanna Lewis, Gerald M Rubin

**Affiliations:** 1 Department of Molecular and Cell Biology, University of California Berkeley, Berkeley, California, United States of America; 2 Howard Hughes Medical Institute, University of California Berkeley, Berkeley, California, United States of America; 3 Department of Genome Sciences, Lawrence Berkeley National Laboratory, Berkeley, California, United States of America; Celera Genomics, United States of America

## Abstract

We have used the annotations of six animal genomes *(Homo sapiens, Mus musculus, Ciona intestinalis, Drosophila melanogaster, Anopheles gambiae,* and *Caenorhabditis elegans)* together with the sequences of five unannotated *Drosophila* genomes to survey changes in protein sequence and gene structure over a variety of timescales—from the less than 5 million years since the divergence of D. simulans and D. melanogaster to the more than 500 million years that have elapsed since the Cambrian explosion. To do so, we have developed a new open-source software library called CGL (for “Comparative Genomics Library”). Our results demonstrate that change in intron–exon structure is gradual, clock-like, and largely independent of coding-sequence evolution. This means that genome annotations can be used in new ways to inform, corroborate, and test conclusions drawn from comparative genomics analyses that are based upon protein and nucleotide sequence similarities.

## Synopsis

Just as protein sequences change over time, so do gene structures. Over comparatively short evolutionary timescales, introns lengthen and shorten; and over longer timescales the number and positions of introns in homologous genes can change. These facts suggest that the intron–exon structures of genes may provide a source of evolutionary information. The utility of gene structures as materials for phylogenetic analyses, however, depends upon their independence from the forces driving protein evolution. If, for example, intron–exon structures are strongly influenced by selection at the amino acid level, then using them for phylogenetic investigations is largely pointless, as the same information could have been more easily gained from protein analyses. Using 11 animal genomes, Yandell et al. show that evolution of intron lengths and positions is largely—though not completely—independent of protein sequence evolution. This means that gene structures provide a source of information about the evolutionary past independent of protein sequence similarities—a finding the authors employ to investigate the accuracy of the protein clock and to explore the utility of gene structures as a means to resolve deep phylogenetic relationships within the animals.

## Introduction

Sequence alignment and comparison have revealed much about evolution at the nucleotide and amino acid level, but much less is known about the structural evolution of genes—how their intron–exon structures, intron lengths, alternative splicing, and untranslated regions change over time. Genome annotations comprise an invaluable resource for answering such questions because they describe the essential parts of a gene and their relationships to one another [1]—information that is missing from protein and transcript sequence files.

Although the origins and mobility of introns are still subjects of debate, previous studies [2,3] have established that just as amino acid sequences change over time, so do gene structures. Over comparatively short evolutionary timescales, introns lengthen and shorten [4]; and over longer timescales the number and positions of introns in orthologous and paralogous genes can change [5]. These facts suggest that the intron–exon structures of genes may provide a novel source of evolutionary information irrespective of the mechanistic details of intron origin and dispersal. Indeed, several studies have already employed them for this purpose [6–8].

The utility of gene structures as materials for phylogenetic analyses, however, depends upon their independence from the forces driving protein sequence evolution. If, for example, intron–exon structures are strongly influenced by selection at the protein level, then using them for phylogenetic investigations is largely pointless, as the same information could have been more easily gained from protein analyses. Also needed is a better understanding of the rates at which different aspects of gene structures evolve. Clearly, more slowly evolving aspects of gene-structure—intron positions [9–11], for example—are best suited to probing deep phylogenetic relationships, whereas more rapidly evolving components—such as intron lengths—are better suited for investigations of more recent events. Here too, comparison to protein evolution is also essential. If intron positions change more rapidly than protein sequences do, their power to resolve ancient relationships will be limited, even if they evolve independently of proteins.

In order to address these issues, we have characterized the number, position, and length of introns and exons in 11 individual genomes representing four phyla. These data provide a panoramic perspective from which to investigate the evolution of gene structures on a variety of timescales—from the less than 5 million years since the divergence of Drosophila simulans and *D. melanogaster,* to the more than 500 million years that have elapsed since the Cambrian explosion.

We show that evolution of intron lengths and positions is largely—though not completely—independent of protein sequence evolution. Thus, gene structures provide a source of information about the evolutionary past independent of protein sequence similarities. We use this fact to investigate the accuracy of the protein clock and to explore the utility of gene structures as a means to resolve deep phylogenetic relationships within the animals.

## Results

### Development of an Open-Source Software Library for Comparative Genomics

In order to facilitate the use of genome annotations as substrates for computational analyses, we developed an open-source software library (CGL) for comparative genomics using genome annotations. The software and a tutorial on its use are available at http://www.yandell-lab.org/cgl.

CGL can convert the annotations from many different databases into a single standardized format; thus the software can be used to assemble very large repositories of annotations that encompass the contents of multiple genome databases. For purposes of the analyses presented here, we have used CGL to convert the genome annotations *of Homo sapiens* [12,13], Mus musculus [14], and Caenorhabditis elegans [15,16] as distributed by GenBank; D. melanogaster annotations from FlyBase [17–19]; the Anopheles gambiae [20] annotations from Ensembl [21]; and the Ciona intestinalis [22] annotations from the JGI [23] into a single standardized file format that greatly facilitates computational analyses. The resulting repository is unique in that no single database or genome project maintains or distributes all of these annotations.

The Bilaterian animals are generally classified as either protostomes or deuterostomes. In deuterostomes, the blastopore lip becomes the anus, whereas in the protostomes it becomes an anterior oral structure. The two lineages are believed to have last shared a common ancestor more than 500 million years ago, and the nematodes may have diverged from both lineages even earlier [24]. We chose the genomes included in this study in such a way as to facilitate inquiries into the evolution of gene structure across various timescales using a minimum number of genomes. Accordingly, we chose to analyze the genomes of three deuterostomes, *H. sapiens, M. musculus,* and *C. intestinalis,* and an equal number of protostomes: *D. melanogaster, A. gambiae,* and C. elegans. This dataset also contains a deep split in both the protostome and deuterostome clades. *C. intestinalis,* a Urochordate, is believed to have diverged from the Craniata—the phylum to which humans and mice belong—about 500 million years ago [25,26]; likewise among the protostomes, D. melanogaster and A. gambiae are believed to have diverged from one another approximately 250 million years ago [26]. The dataset thus contains a number of deeply divergent animal genomes, making it ideal for the investigation of long-term trends in the evolution of gene structures. Inclusion of the human and mouse genomes makes possible investigations of more rapidly changing aspects of gene structure, as they are believed to have diverged from one another about 70 million years ago [14].

In order to survey gene evolution during even shorter time intervals, we also included in our dataset five recently sequenced but unannotated genomes: D. simulans [27], D. yakuba [27], D. ananassae [28], D. pseudoobscura [29], and D. virilis [28]. These five *Drosophila* species are believed to have diverged from the *melanogaster* lineage around 5 million, 13 million, 44 million, 55 million, and 63 million years ago, respectively [30]. Because CGL can extract a wide array of information pertaining to the evolution of gene structure even from incompletely assembled and unannotated genomes, this effectively gave us a dataset of 11 genomes for our analyses. The inclusion of these provisionally assembled and unannotated genomes also allowed us to examine the utility of unfinished genomes for analyses of gene evolution.

### Intron–Exon Structure in Six Animal Genomes

As our collection of annotated genomes contained more than 100,000 annotations, we sought first to survey and summarize the contents of each genome's annotations with regards to gene structure. We choose three basic measures: intron length, exon length, and intron density. These measures provide a concise summary of the similarities and differences in intron–exon structure for the six annotated genomes. Placing these data in their phylogenetic context allows trends in the evolution of gene structure to emerge.

#### Intron length.

**Figure 1:**
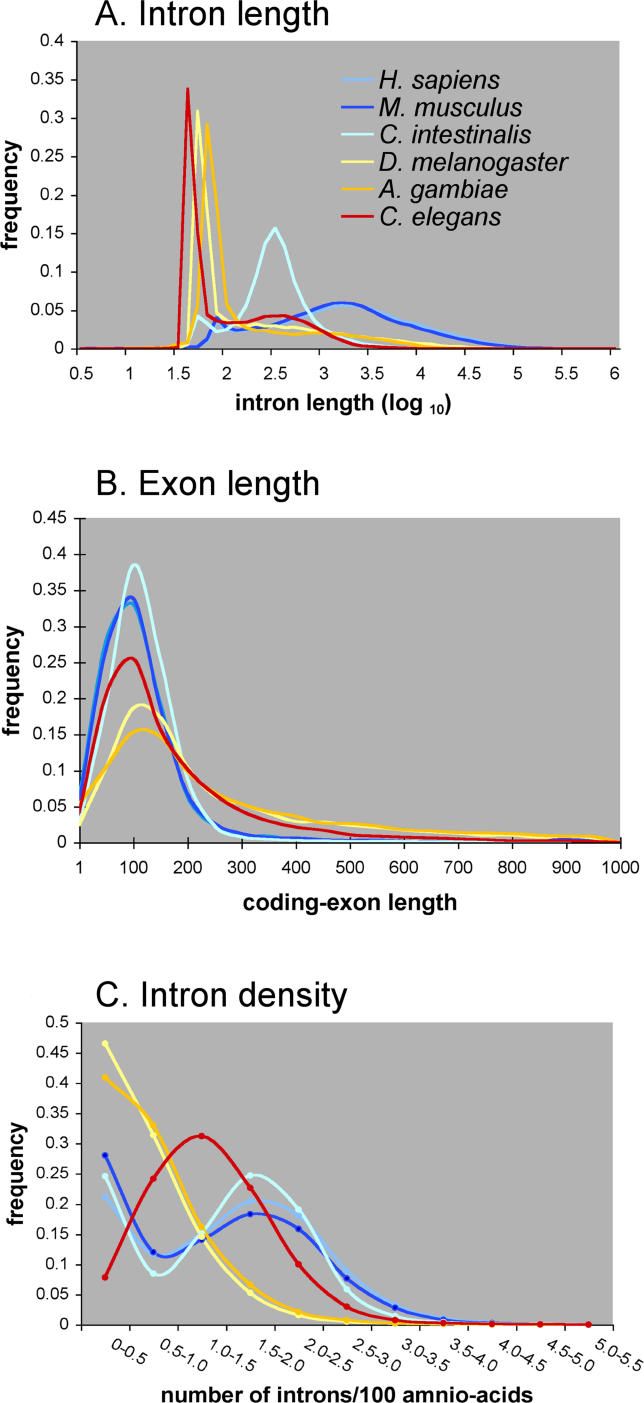
Global Overview of Gene Structure in Six Annotated Animal Genomes (A) Intron length. Annotated intron length (log_10_) is plotted on the *x*-axis; the frequency at which introns of that length occur in an organism's genome is plotted on the *y*-axis. (B) Exon length. *x*-axis, coding-exon length in nucleotides; *y*-axis, frequency. (C) Intron density. A transcript's intron density is equal to its number of coding introns divided by the length of the protein it encodes. *y*-axis, frequency of annotated transcripts with a particular intron density. *x*-axis, intron density binned in increments of 0.5 introns/100 amino acid (see Materials and Methods). Deuterostomes are shown in shades of blue; protostomes in shades of red.

Figure 1A shows the distribution of intron lengths in each of the six annotated genomes. The fact that the D. melanogaster distribution is bimodal has been noted before, and used to classify its introns based upon their lengths [31]—here termed class-I (<100 nucleotides [nt]) and class-II (>100 nt). Two trends emerge when these genomes are compared. First, there is a pronounced shift in modes between the deuterostome and protostome distributions. Indeed, the relative proportion of introns falling into either intron class is more or less reversed between the two animal clades; most protostome introns are class-I, whereas most deuterostome introns are class-II. Second, introns are not merely longer in the deuterostomes; instead, the shift toward longer introns in these genomes appears to have occurred at the expense of introns of intermediate length. These data suggest that selection on intron lengths may be bimodal, with variability in the lengths of the two modes due to differences in the lengths and numbers of transposons characteristic of each genome.

#### Exon length.

We also characterized each genome with respect to coding-exon length (Figure 1B). All six exon-length distributions have similar modes. Mean coding-exon lengths, on the other hand, differ substantially between the protostome and deuterostome genomes. The mean coding-exon length is 149, 149, and 152 nt in the human, mouse, and C. intestinalis genomes, respectively, whereas it is 364, 365, and 210 nt in the *D. melanogaster, A. gambiae,* and C. elegans genomes, respectively. The longer means associated with the protostome distributions reflect the general paucity of introns in these genomes (see following section on intron density). The fact that all six exon-length distributions have such similar modes, despite their dissimilar mean lengths, suggests that that intron spacing may be under similar selective pressure in all six genomes.

**Figure 2:**
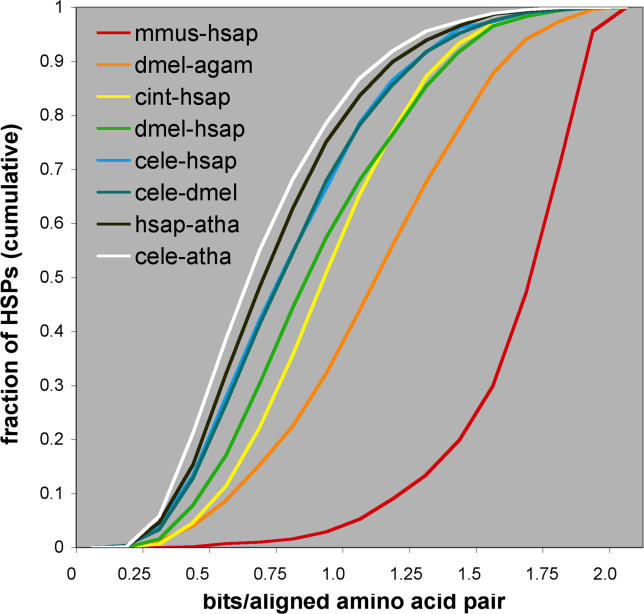
Cumulative Distribution Functions Illustrating Proteome-Wide Trends in Protein Similarity *x*-axis, bits/aligned position; *y*-axis, cumulative fraction of HSPs having that number of bits/aligned amino acid pair or less. To facilitate display, only a subset of the 21 possible pair-wise combinations is shown. Data are based upon all reciprocal best BLASTP hits identified in all versus all BLASTP searches of the proteomes. Similarity calculations were restricted to the high-scoring HSP for each BLAST hit, in order to avoid data duplication due to overlapping alignments. There were 13,339 *M. musculus–H. sapiens* reciprocal best hits; 6,435 between D. melanogaster and *A. gambiae;* 5,828 between C. intestinalis and *H. sapiens;* 5,542 between D. melanogaster and *H. sapiens;* 4,669 between C. elegans and *H. sapiens;* 4,588 between C. elegans and *D. melanogaster;* 3,361 between H. sapiens and *A. thaliana;* and 2,835 between C. elegans and A. thaliana. atha, *A. thaliana;* cele, *C. elegans;* cint, *C. intestinalis;* dmel, *D. melanogaster;* hsap, *H. sapiens;* mmus, *M. musculus.*

One process that might explain the longer exons characteristic of the protostome genomes is retro-transposition-mediated gene duplication [32]. Because this process results in intronless copies of existing genes, each event will tend to stretch the distribution of exon lengths, shown in Figure 2 toward the right. Although the two insect genomes are enriched for intronless genes as compared to human and mouse, this is not true of the C. elegans genome. This fact argues against such a process. Thus, these data support a model whereby introns are being lost (or gained) from genes in piecemeal fashion within all six genomes.

#### Intron density.

In order to further investigate the distribution of introns, we have made use of a simple summary statistic of gene structure: intron density, or the number of coding introns associated with a particular protein divided by that protein's length [33]. Although in principle, genome-wide fluctuations in protein lengths might also affect this measure, this does not appear to be the case. To control for this possibility, we recalculated the intron densities for each of the six genomes, using only conserved portions of each annotated protein (unpublished data); the resulting distributions (discussed below) were essentially unchanged, demonstrating that changes in intron density reflect differences in intron numbers, not changes in protein lengths. Intron density thus provides a precise definition with which to distinguish intron-rich from intron-poor genes.

While intron density is an attribute of a single annotated transcript, when applied to entire annotated genomes it can also be used to provide a summary statistic regarding the distribution of introns within a genome. Consistent with the exon-length distributions shown in Figure 1B, the three deuterostome genomes are more intron-rich than the protostome genomes. Deuterostome mean intron-densities are 1.53, 1.47, and 1.42 introns/100 amino acids in the human, mouse, and C. intestinalis genomes, respectively; and 1.21, 0.61, and 0.58 for the *C. elegans, D. melanogaster,* and A. gambiae genomes, respectively.

To explore these data more closely, we also examined the frequency distributions of intron density in each of the six annotated genomes (Figure 1C). As was the case for intron length, the deuterostome intron-density distributions are bimodal; thus these genomes contain two classes of annotated genes: one intron-rich, the other intron-poor. This, however, may be partially a consequence of pseudo-genes having been annotated as genes in these genomes, as the intron-poor peak largely (but not entirely) disappears if intronless genes are excluded from the calculation. By comparison to the deuterostome genomes, most insect genes are intron-poor. The C. elegans distribution is unimodal and distinctively different from those of the other five animals.

The data in Figure 1C make it clear that the ratio of intron insertion to deletion has varied in a lineage-dependent fashion in the course of animal evolution. Moreover, the shape of the A. gambiae and D. melanogaster distributions suggests that there may be a long-term trend in the *Diptera* toward intron loss. This process of intron loss appears to have occurred in a largely piecemeal fashion: when intronless genes are excluded from the calculation, the resulting dipterian intron density distributions are essentially unchanged from those shown in Figure 1C. We also examined the intron density distribution of the Apis mellifera genome. Its distribution (unpublished data) is similar to that of *C. elegans,* a finding also consistent with large-scale intron loss in the *Diptera*.

No matter what the ancestral animal distribution may have looked like, the diversity of the present-day intron density distributions makes it certain that extensive remodeling of intron–exon structures has occurred in at least some of these genomes since the six animals last shared a common ancestor. Several lines of evidence suggest that this process has been a slow one. Current estimates of the rate of intron insertion and deletion in animal genomes have placed it at less than one event/gene/200 million years [11]. Each of the animal genomes in our study contains tens of thousands of introns; this fact, together with the low intron indel rate, means that a vast period of time will have to elapse before any fluctuation in the ratio of intron insertion to deletion will act to alter the global distribution of introns within a genome. Intron density distributions are thus likely to be among the more slowly evolving attributes of any animal genome. The two insect distributions serve well to illustrate this point: their intron density distributions (Figure 1C) mirror each other to a remarkable degree despite nearly 250 million years of independent evolution [26].

### A Survey of Proteome-Wide Patterns of Protein Similarities

Next, we sought to characterize and compare the six annotated proteomes to one another with respect to protein similarities. These analyses are a necessary prerequisite for an examination of intron–exon structures in the context of protein similarities. As a first step, we preformed an all-against-all BLASTP [34] search of the six annotated proteomes, and recovered sets of pair-wise reciprocal best hits. From each BLASTP hit, we then selected the highest-scoring high-scoring segment pair (HSP) to avoid complications arising from overlapping sequence alignments. These reciprocal best-hit best HSPs provide nonidentical but intersecting sets of putative orthologs with which to examine patterns of protein evolution. A strength of this approach is that it makes available the largest possible set of putative orthologs for subsequent analyses. This means that gene families restricted to a subset of the proteomes will be included, as will more rapidly evolving proteins that lack clear orthologs over all evolutionary distances. Thus, the analyses presented below provide an overview of protein similarities on the largest possible scale, and complement previous analyses employing smaller subsets of orthologous proteins drawn from different combinations of annotated proteomes [35–37].

#### Proteome-wide patterns of similarity.

Figure 2 summarizes the distribution of pair-wise similarities among these putative orthologs for selected pairs of proteomes. The figure uses bits/aligned amino acid pair as a similarity measure. This information-theoretic measure of similarity [38,39] is obtained by dividing the bit score of a BLASTP alignment by its length. The measure is proportional to percent identity and percent similarity but is more precise.

Figure 2 provides a means to examine the extent to which the similarities among the annotated proteomes reflect phylogeny [24,26,40]. For example, 75% of the high-scoring reciprocal best-hit best BLASTP HSPs identified in an all-by-all comparison of the human and mouse proteomes contain more than 1.5 bits/aligned position; whereas only 3% of the HSPs recovered from a comparison of the human and D. melanogaster proteomes contain more than 1.5 bits/aligned position. The numbers of reciprocal best-hit best HSPs recovered from each pair-wise comparison (see Figure 2 legend) are also consistent with phylogeny.

In order to assay the impact of unequal rates of protein evolution on these data, we also compared the six animal proteomes to the A. thaliana [41] proteome. Previous studies of C. elegans 18s ribosomal sequences and proteins have suggested that they are rapidly evolving [40,42], and our data demonstrate that this is also the case for the proteome as a whole: C. elegans reciprocal best-hit best HSPs are consistently less similar to their *Arabidopsis* partners than are human and A. thaliana HSPs (Figure 2). None of the other genomes were found to be particularly rapidly evolving by this assay.

### Recasting Trends in Protein Similarity as a Phylogenetic Tree

**Figure 3:**
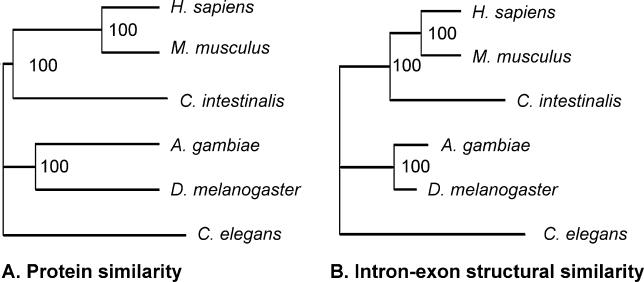
Neighbor-Joining Trees Summarizing Proteome-Wide Trends in Protein Similarity and Genome-Wide Trends in Intron–Exon Structural Similarity Proteome-wide trends in protein similarity (A), and genome-wide trends in intron–exon structural similarity (B). Numbers beneath tree nodes are bootstrap values.

For purposes of further analysis, we recast the distributions shown in Figure 2 into a phylogenetic tree. To do so we concatenated every reciprocal best-hit best HSP derived from each pair-wise proteome–proteome comparison and calculated the bits/aligned amino acid pair for the resulting concatenated alignment. We used that value as a measure of inter-proteome similarity, and built a 6 × 6 distance matrix from which we constructed a phylogenic tree (see Materials and Methods). The resulting tree is shown in Figure 3A.

This approach to consensus phylogenetic tree construction differs from standard methods [43] in that it bypasses the need to construct multiple alignments as a prelude to tree construction; thus, it is much faster than existing approaches, and scales well for comparisons of multiple annotated proteomes. An additional strength of the approach is that it lends itself in a natural fashion to bootstrap analysis [44]. Bootstrap values for each node in the tree can be obtained by randomly and repeatedly resampling a subset of the HSPs from each pair-wise comparison of proteomes, constructing a new tree using these data, and then ascertaining how frequently the resulting trees contain the same nodes as the consensus tree (see Materials and Methods for more details). As the bootstrap values in Figure 3A demonstrate, proteome-wide patterns in similarity (some of which are shown in Figure 2) are consistent with only a single phylogenetic tree. The fact that the resulting tree is robust and consistent with phylogeny provides a benchmark by which to judge the success of this approach to tree building, and demonstrates its utility as a means to summarize trends in large quantities of similarity data.

### Intron–Exon Structures in the Context of Protein Similarities

Our characterization of proteome-wide patterns of amino acid similarities (summarized in Figures 2 and 3A) provides an ideal context within which to investigate the evolution of intron–exon structures. Because CGL provides an easy means to simultaneously investigate amino acid similarities and intron–exon structures, we were able to investigate genome-wide trends in intron–exon structures using the very same HSPs that we used for our analyses of protein similarities.

Cursory examination of these HSPs makes clear two important facts. First, genome-wide trends in intron–exon structural similarities roughly parallel those of phylogeny and protein similarity. For example, 92% of human–mouse, 36% of human–*C. intestinalis,* and 15% of human–D. melanogaster reciprocal best-hit best HSPs have identical intron–exon structures. Summarizing similarities in intron–exon structures as simple percentages, however, fails to account for the fact that intron densities vary between genomes. As our earlier characterization of intron densities revealed (Figure 1C), the D. melanogaster genome is relatively intron poor: its mean intron density is less than half that of the C. elegans and human genomes. Not correcting for this factor introduces a bias into the percentages, and this fact must be considered when subjecting these data to more rigorous analyses.

#### Quantifying similarities in intron–exon structures.

In order to address differences in intron density, we formulated a more exacting, though less intuitive, definition of intron–exon structural similarity that takes intron density into account. To do so, we calculated a log odds ratio (LOD) score for each set of concatenated reciprocal best-hit best HSPs in toto, wherein the ratio of the observed number of aligned splice junctions to the expected frequency was used as a measure of global similarities in intron positions for two genomes. To obtain the expected frequency of aligned introns, we multiplied the frequencies of introns within query and subject portions of the concatenated alignment. Thus this measure of intron–exon similarity controls for the differing frequencies of introns in the different genomes. It is also essentially identical to the standard LOD score approach used to measure protein similarities [45].

To summarize the results of this analysis, we recast the resulting matrix of LOD scores into the phylogenetic tree shown in Figure 3B. Our approach to constructing the tree was identical to the one used to produce Figure 3A, and consisted of once again concatenating every reciprocal best-hit best HSP derived from each pair-wise proteome–proteome comparison; only this time, rather than quantify amino acid similarities, we instead calculated the intron–exon LOD score for the resulting concatenated alignment. We then used that value as a measure of intron–exon structural similarity between two genomes, and built a 6 × 6 distance matrix from which we constructed a bootstrapped phylogenic tree (see Materials and Methods).

As was the case for protein similarities (Figure 3A), genome-wide trends in gene structures (Figure 3B) are also consistent with a single tree whose topology mirrors phylogeny. Because both trees were constructed using exactly the same HSPs, the two trees shown in Figure 3 naturally complement one another: Figure 3A summarizes the proteome-wide patterns in amino acid similarities, Figure 3B genome-wide similarities in the intron–exon structures of those very same annotated genes. Although the jagged right-hand side of the tree suggests that the forces governing change in gene structure are less uniform than those governing protein evolution, Figure 3B demonstrates that genome-wide patterns in intron–exon structures parallel those of protein similarities.

### Intron–Exon Structures Evolve Independently of Protein Sequences

#### Protein identity versus intron–exon structure.

**Figure 4:**
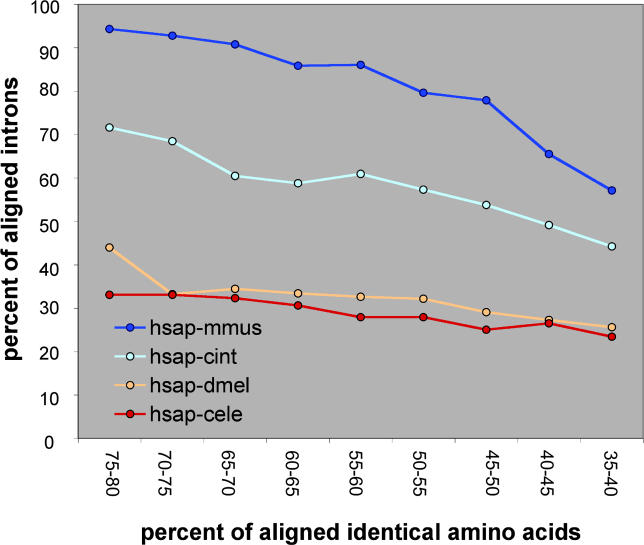
Intron–Exon Structures Evolve Largely Independently of Protein Sequences *x*-axis, human reciprocal best-hit best HSPs for four representative proteomes binned by percent identity in 5% increments. *y*-axis, percent of aligned introns among the HSPs in each bin. cele, *C. elegans;* cint, *C. intestinalis;* dmel, *D. melanogaster;* hsap, *H. sapiens;* mmus, M. musculus.

One issue not addressed by our previous analyses is the extent to which evolution of intron–exon structures is coupled to that of protein sequences. A clear understanding of the impact of protein-sequence evolution on gene structures is desirable if gene structures are to be used for phylogenetic investigations. Figure 4 demonstrates that a significant portion of intron–exon structural evolution is proceeding independently of protein conservation. Among human–mouse reciprocal best-hit best HSPs with 50%–55% amino acid identity, 80% of their introns are aligned to one another. The percentage declines to 57% among human–*Ciona* reciprocal best-hit best HSPs having the same amino acid identity, and it drops still further for human–D. melanogaster and human–C. elegans reciprocal best-hit best HSPs. This trend is clearly consistent with phylogeny, and holds true regardless of the degree of amino acid conservation. Thus the y-intercept of the curves in Figure 4 is an indicator of phylogenetic distance.

Although phylogeny is the primary factor structuring the data in Figure 4, it nonetheless appears that intron–exon structures and protein sequences do not evolve entirely independently of one another. If protein sequence conservation had no impact on gene structures, then each of the curves in Figure 4 would be straight, horizontal lines, and the differences in their y-intercepts would reflect time since last common ancestor. In actual fact, the negative slopes of the curves imply that protein sequence conservation is acting to slow the evolution of intron–exon structures. Among human–mouse reciprocal best-hit best HSPs having 65%–70% amino acid identity, 90% of their introns are aligned, whereas fewer than 70% of introns are aligned in human–mouse reciprocal best-hit best HSPs having 40%–45% amino acid identity. Similar trends occur in human–*Ciona,* human–*D. melanogaster,* and human–C. elegans reciprocal best-hit best HSPs. These facts show that genes encoding highly conserved proteins tend to have more similar intron–exon structures than those encoding less well-conserved proteins. Thus, it appears that protein sequence conservation is acting to slow the rate at which intron–exon structures change. It should be kept in mind, however, that some portion of this trend may be due to the power of the reciprocal best-hit approach to identify orthologous genes, and the accuracy of the protein alignments themselves. Less well-conserved proteins may be less accurately aligned to one another than highly conserved proteins, with the consequence that their gene structures also appear less well conserved. On the balance then, the data in Figure 4 suggest that a significant portion of intron–exon structural evolution is proceeding independently of protein conservation, though protein sequence conservation is acting to slow its rate.

#### Controlling for the impact of protein conservation.

**Figure 5:**
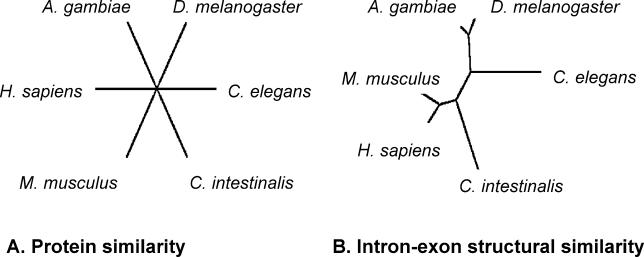
Controlling for the Impact of Unequal Rates of Protein Evolution on the Evolution of Intron–Exon Structures (A) Unrooted neighbor-joining tree based upon amino acid similarities for reciprocal best-hit best HSPs having 1.25 bits/aligned amino acid pair. (B) Unrooted neighbor-joining tree based upon similarities in the intron–exon structures of those same HSPs.

The finding that the rate of change in a gene's intron–exon structure is influenced by selection on the protein it encodes (Figure 4) means that the phylogenetic trees produced using intron–exon structures (Figure 3B) and proteins (Figure 3A) are not entirely independent of one another. Thus, we sought to discover to what extent—if any—proteome-wide trends in protein similarity influenced the topology of the tree shown in Figure 3B. To answer this question, we developed a means to construct a phylogenetic tree of intron–exon structures free of this complication. To do so, we made use of the same procedure we used to construct Figure 3B; only this time, we restricted the reciprocal best-hit best HSPs used to construct each pair-wise concatenated alignment such that the overall amino acid similarity of each concatenated alignment was fixed at a preselected value of 1.25 bits/aligned amino acid pair (see Materials and Methods). This restriction allowed us to control for the braking effect (Figure 4) exerted by protein sequence conservation on the evolution of intron–exon structures, as in each case the resulting pair-wise concatenated alignments had exactly the same degree of protein sequence conservation (approximately 50% identity). However, because no such restriction was placed on them with respect to similarities in their intron–exon structures, they were free to vary in this regard. The resulting unrooted tree based upon protein similarities alone is shown in Figure 5A—its star topology is a consequence of our having held protein similarities constant. The tree shown in Figure 5B was constructed from the same HSPs as the tree in 5A, only this time using intron–exon structural similarities as measured by the same metric used to produce the tree shown in Figure 3B.

Note that the tree in 5B suggests the same phylogenetic relationships as the tree shown in Figure 3B; this fact has two implications. First, the unequal terminal branch lengths of the tree in Figure 5B demonstrate that when protein conservation is controlled for, rates of change in intron–exon structures remain unequal—no matter what taxon is chosen as a root, the resulting tree will retain the jagged right-hand edges characteristic of the tree shown in Figure 3B. This fact demonstrates that lineage-specific processes play an important role in determining gene structures independent of any lineage-specific trends in the rates of protein evolution, such as the rapid evolution of C. elegans proteins. Second, the fact that the trees shown in Figures 5B and 3B have identical topologies means that the braking effect exerted by protein conservation upon the rate of change of intron–exon structures (Figure 4) has had little impact on their evolution at the genome scale. This means that intron–exon structures provide a source of phylogenetic information independent of protein evolution.

### Evolution of Intron Lengths

#### The quartet dataset.

Having examined the evolution of intron–exon-structures, we next sought to investigate the evolution of intron lengths. Previous work [46] in this area has shown that similarities in intron lengths can be used for phylogenetic analyses. Our analyses further characterize the evolution of intron lengths. As a first step toward these investigations, we used a reciprocal best-hit approach to identify sets of human and mouse orthologous paralog pairs that we term “quartets.” Each quartet consists of four genes: a pair of human paralogs and their corresponding mouse orthologs, all of which share the same intron–exon structure as judged by the positions of their annotated splice junctions relative to the protein alignments of their reciprocal best-hit best HSPs. In total, we were able to identify 1,265 quartets. Note that every quartet is in theory the product of the same historical process—some gene duplicated before the time humans and mice last shared a common ancestor, and the products of this duplication event are represented today by genes *i* and *j* in the human genome and *i*′ and *j*′ in the mouse genome. This implies that the time since speciation will be less than (or equal to) the time since duplication. Hence, the orthologous members of a quartet share a more recent common ancestor than do the paralogous members of a quartet.

#### Vertebrate intron pairs.

**Figure 6:**
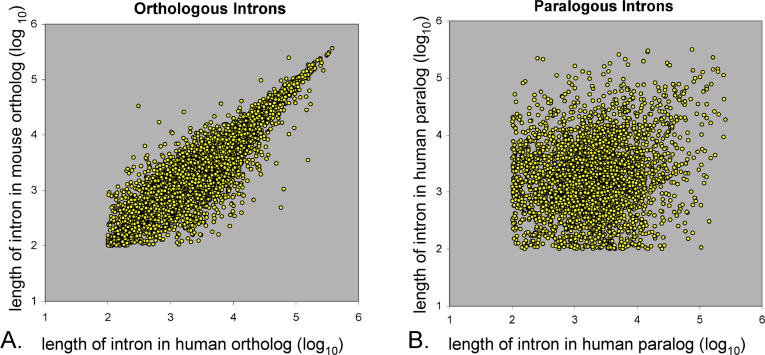
Lengths of Orthologous Introns within a Quartet Are More Correlated than Those of Paralogous Introns (A) Quartet orthologous intron pairs. *x*-axis, length (log_10_) of introns in human members of each quartet; *y*-axis, length (log_10_) of corresponding orthologous introns in the mouse member of the same quartet. Spearman correlation coefficient: 0.903; *p* < 0.001. (B) Paralogous introns. *x*-axis, length (log_10_) of introns in human members of each quartet; *y*-axis, length (log_10_) of corresponding paralogous introns in the other human member of the same quartet. Spearman correlation coefficient: 0.140; *p* < 0.001. The mouse distributions are essentially identical to their human counterparts.

As Figure 6 demonstrates, intron lengths among orthologous quartet members (Figure 6A) are much more correlated with one another than are those of paralogous quartet members (Figure 6B). Why might this be so? Under the assumption that the paralogous introns of newly duplicated genes are usually the same length, and that over time the correlation in their lengths declines, the results in Figure 6 are consistent with our hypothesis that orthologous quartet members share a more recent common ancestor than do paralogous quartet members.

If this interpretation is correct, then the data in Figure 6 have something to say about the rate of intron length evolution. First, they make it clear that orthologous intron lengths can remain correlated for considerable periods of time following speciation events. Despite that fact some 70 million years have elapsed since humans and mice last shared a common ancestor, orthologous intron lengths are still correlated in these two organisms. Likewise, the weaker correlation characteristic of paralogs (Figure 6B) is consistent with the duplication events producing these paralogous genes having occurred much more than 70 million years ago.

#### A possible clock.

The data in Figure 6 suggest that changes in intron lengths might be used as a clock to date speciation and gene duplication events. These data, however, are inadequate to either prove or disprove such a hypothesis. Although it is possible to think of the results in Figure 6 as the result of a decay process whereby orthologous intron lengths following speciation events, and paralogous introns lengths following duplication events, gradually become less similar to one another over time, showing that this is actually the case requires intermediate time points.

**Figure 7:**
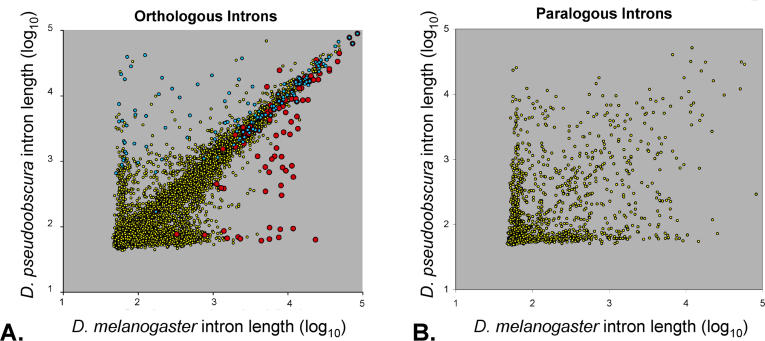
D. melanogaster Intron Lengths Are Highly Correlated with Their Inferred D. pseudoobscura Orthologs; D. melanogaster Paralogous Introns Show No Such Correlation (A) *x*-axis, length (log_10_) of annotated D. melanogaster introns; *y*-axis, length (log_10_) of their inferred orthologs in the D. pseudoobscura genome. Red circles indicate those introns containing a transposon in *D. melanogaster;* blue circles indicate those introns containing a transposon in *D. pseudoobscura;* gold circles indicate introns without identifiable transposons in either species. Spearman correlation coefficient: 0.637; *p* < 0.001. (B) Intron lengths of paralogs having the same intron–exon structure as judged by the positions of their splice junctions relative to the protein alignments of their reciprocal best-hit best HSPs. *x*-axis, length (log_10_) of introns in an annotated D. melanogaster gene; *y*-axis, length (log_10_) of corresponding paralogous introns. Spearman correlation coefficient: 0.448; *p* < 0.001.

To further investigate these questions, we turned to the six *Drosophila* genomes in our collection. Unfortunately, none of the recently sequenced *Drosophila* genomes has yet been annotated. Thus we could not use the reciprocal best-hits approach we used to explore correlations in intron lengths in Figure 6. Instead, we used TBLASTN [34] to identify the coordinates of each annotated D. melanogaster protein's best hit in each of the five unannotated *Drosophila* genomes, and CGL to infer the positions of putative orthologous introns using the resulting TBLASTN HSPs. The results of this approach using the D. pseudoobscura genome are shown in Figure 7A.

As can be seen, the lengths of the inferred D. pseudoobscura orthologous introns are highly correlated with their D. melanogaster partners, despite 55 million years of independent evolution. These results show that, in both vertebrates and insects, orthologous intron lengths can remain correlated over tens of millions of years following speciation events, despite the different distributions of intron lengths (Figure 1), generation times, and population dynamics of the deuterostome and protostome clades.

### Forces Shaping Correlations in Intron Lengths

The two distributions of orthologous intron pairs shown in Figures 6 and 7, however, do differ in several significant details. The insect distribution has an abundance of pairs located at the origin and stretching along the *x*- and *y*-axes, producing an arrowhead-like structure at the graph's origin (Figure 7A). The vertebrate distribution (Figure 6A), lacks this feature, and instead is characterized by a central bulge. In order to better understand these features of the two distributions, we examined the repeat content of the orthologous intron sets using RepeatMasker [47].

In general, the transposon load of the vertebrate introns is higher than that of the insects, and much of the central bulge is due to the presence of additional LINE elements in either the human or mouse member of the pair (unpublished data). This is in sharp distinction to the two insects. Although some of the larger off-diagonal intron pairs in the insect distribution (Figure 7A) contain a transposon in one or both members of the pair, in general there are far fewer transposons in the insect introns, and there are no cases wherein both members of an insect–intron pair contain the same transposon, an event that is quite common among human–mouse pairs.

Although transposons seem to explain the central bulge in the human–mouse distribution, they do not explain the details of the *melanogaster–pseudoobscura* distribution, since most of the intron pairs that comprise the arrowhead-like portion of the insect distribution are entirely transposon-free. Simple repeats and repetitive sequences also do not appear to play an important role in structuring this portion of the distribution, as there is no obvious tendency for the longer partner of the pair to contain additional low-complexity sequences (unpublished data). We also investigated the possibility that the arrowhead region might be an artifact of the assembly process. Although it is difficult to rule out this possibility, gaps in the D. pseudoobscura assembly did not seem especially over-represented in this portion of the distribution; moreover, given the mature state of the D. melanogaster assembly, it is inconceivable that there is sequence missing from a large number of D. melanogaster introns. Instead, we believe that the arrowhead-like portion of the insect distribution shown in Figure 7A is simply a consequence of selection pressures for short intron lengths in the *Drosophilae*. Certainly, the tight distribution of insect intron lengths shown in Figure 1 will guarantee that an intron of any length in one *Drosophila* species will tend to be paired with an ortholog in another species whose length lies near the mode, a fact that will tend to produce the arrowhead portion of distribution shown in Figure 7A. This hypothesis may also explain why the distributions of human–mouse orthologous introns shown in Figure 6 lack the arrowhead, as the variance in intron lengths is much greater in these two vertebrate genomes (Figure 1A).

The preceding observations imply that the rate at which intron pairs leave the diagonal in Figures 6 and 7 will be influenced by the global distribution of intron lengths within a genome. Attempts to use orthologous intron length correlations as a clock to date speciation events must address this issue, as accurate time estimates will be obtained only if the distribution of intron lengths is similar for every genome under examination. To address this point, we used CGL to determine the intron length distribution for each of the six *Drosophila* genomes in our collection, and found it to be essentially the same in all of them (Figure S1).

No doubt, other less easily measured factors also affect the rate at which intron lengths evolve within a species. If transposon load and/or rates of transposition, for example, vary greatly within two genomes, correlations in the lengths of orthologous introns will be a poor indicator of time since last common ancestor. Rather than attempt to measure the impact of a host of factors on intron lengths, we chose instead to ask a related question. Namely, how constant is the decline in the correlation in orthologous intron lengths over time? Doing so allowed us to directly assess not only whether hypothetical differences in transposition rates actually do act to modify the rates at which length correlations among homologous *Drosophila* introns decline with time, but also if any other factors that we have failed to consider thus far might influence the process as well.

**Figure 8:**
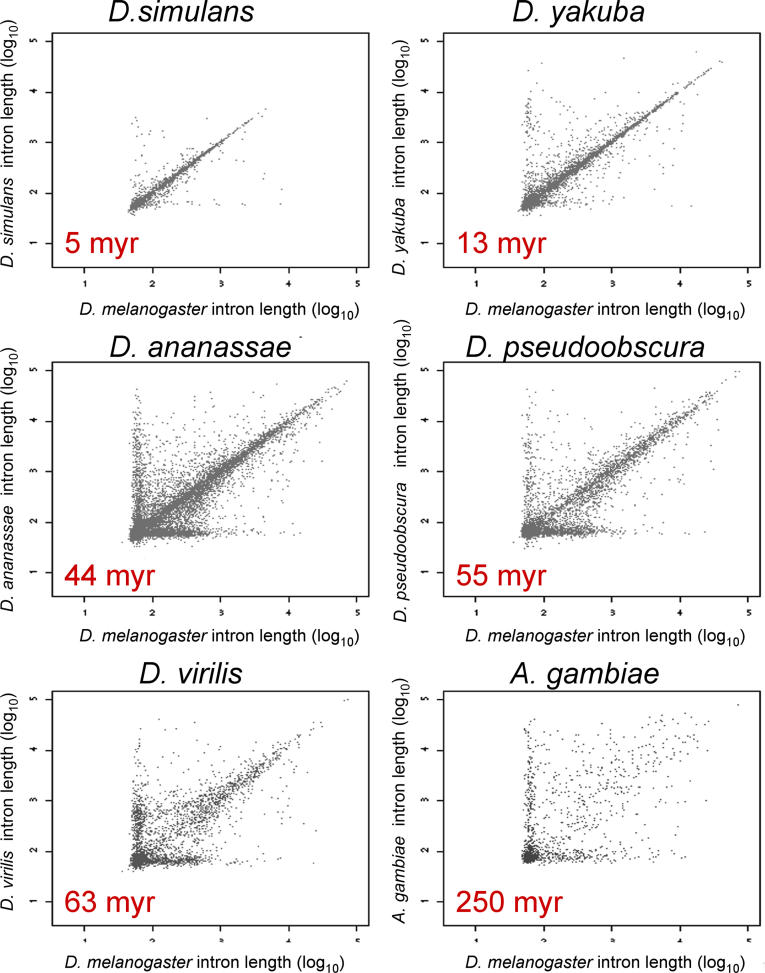
Correlation in Orthologous Intron Lengths Is Proportional to Time since Last Common Ancestor From left to right, and top to bottom: Annotated D. melanogaster lengths (*x*-axis) versus inferred orthologous intron lengths (*y*-axis) for D. simulans (strain 6), *D. yakuba, D. ananassae, D. pseudoobscura,* and D. virilis. Bottom right-hand panel: Annotated D. melanogaster lengths (*x*-axis) versus inferred A. gambiae intron lengths (*y*-axis). Approximate time since last common ancestor is shown in red in the lower left-hand corner in each panel; these are approximate estimates based upon protein data [30]. Spearman correlation coefficients: 0.886, 0.863, 0.670, 0.637, 0.550, and 0.410 for *D. simulans, D. yakuba, D. ananassae, D. pseudoobscura, D. virilis,* and A. gambiae distributions, respectively. *p* < 0.001 for each correlation coefficient. See Materials and Methods for analysis details.

Figure 8 shows the length distributions of inferred orthologous intron pairs for D. melanogaster and each of the five other *Drosophila* species in our collection of genomes. We also performed the same analysis using the A. gambiae genome (lower right-hand panel). Although not entirely free of assembly artifacts—the *melanogaster–simulans* panel, for example, is truncated at about 10,000 base pairs due to short D. simulans contig lengths—Figure 8 nevertheless suggests that intron length evolution has a clock-like nature, as the fraction of intron pairs located along the diagonal in each panel is roughly proportional to the time since last common ancestor [30]. Orthologous intron lengths are highly correlated in the D. simulans and D. yakuba panels, less so in D. pseudoobscura and *D. virilis,* and much less so in *the A. gambiae* panel suggesting that most of the correlation in intron lengths is lost after between 60 million and 250 million years of independent evolution. Moreover, the fact that the magnitudes of the pair-wise correlations in intron lengths mirror established phylogenetic relationships within the *Drosophilae* (Figure S2) provides additional evidence for a strong and consistent historical signal in these data.

#### Intron lengths and the protein clock.

Correlations in orthologous intron lengths seem to accord well with the passage of time (Figure 8). In order to examine this trend more rigorously, we took advantage of the fact that the best-published estimates of time since last common ancestor among the six *Drosophilae* are based upon the protein clock [30]. This allowed us to compare correlations in orthologous intron lengths to the rate of protein evolution. Numerous studies have shown the protein clock to be reasonably constant [48]. Thus, a poor correlation between the two would imply an inconstant rate of change in intron lengths; a strong correlation, a constant rate of change—thereby making it possible to assess the extent to which forces other than the passage of time affect correlations in orthologous intron lengths.

**Figure 9:**
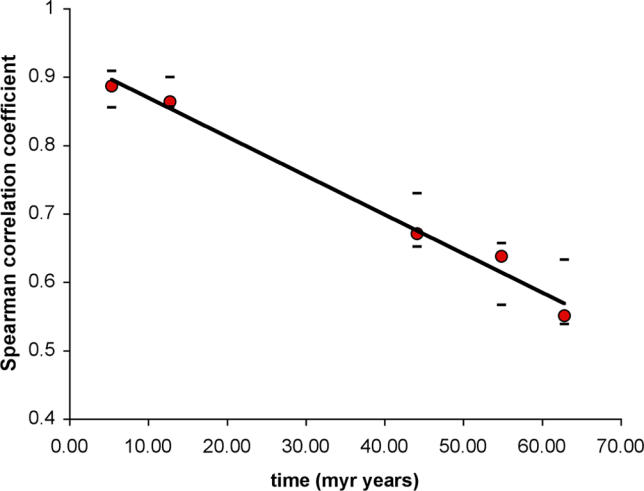
Intron Lengths Can Be Used as a Molecular Clock *y*-axis, magnitude of the Spearman correlation coefficient for the five *Drosophila* distributions shown in Figure 8. *x*-axis, time (millions of years) since last common ancestor based on protein similarities as calculated in [30]. Black bars above and below each data point denote observed variance in the data and were obtained by randomly resampling 1,000 orthologous intron pairs 100 times. Best-fitting curve (shown in black) y = −0.0057x + 0.9266; R^2^ = 0.9875.

Figure 9 plots the Spearman correlation coefficient [49] associated with each of the panels in Figure 8 versus the time since D. melanogaster and one of the five other *Drosophilae* last shared a common ancestor as estimated by the protein clock. As can be seen, there is a strong and constant correlation between the two variables. This fact suggests that the impact of differences in optimal intron lengths, transposon demographics, and rates of transposition on these data is small. Thus, it seems that the passage of time is the dominant force structuring the distributions shown in Figure 8, and that one can speak of an intron clock.

The strong correlation between the intron and protein clocks demonstrated in Figure 9 naturally raises the question as to the extent to which the two are mechanistically coupled. Might selection on flanking exons act to slow the rate at which the lengths of orthologous introns diverge from one another following speciation events? In order to address this issue, we investigated the extent to which correlations between the lengths of orthologous D. melanogaster and D. yakuba introns reflect the intensity of selection on the portion of the protein encoded by exons flanking those introns. To do so, we looked for a correlation between similarities in the lengths of orthologous intron pairs and the average K_a_/K_s_ ratio [50,51] of their flanking exons (Figure S3). We found no significant correlation between the two variables. Thus, the intron length and protein similarity clocks run largely independently of one another.

## Discussion

### Intron Lengths

Our investigations of intron length evolution focused on discovering the forces driving changes in intron lengths; the rate at which they change, whether or not the rate is constant; and if so, over what duration and phylogenetic scope. Intron lengths vary greatly among the six annotated genomes, yet when placed in their phylogenetic context general trends emerge. Every deuterostome genome in our collection is characterized by a predominance of class-II (>100 nt) introns, whereas class-I (<100 nt) introns predominate in the protostome genomes. The similarity in the human and mouse distributions suggests that these distributions change slowly over periods of tens of millions of years. Our examinations of intron lengths within the *Drosophilae* support the same conclusion. Moreover, these data suggest that introns do not simply grow longer and shorter over evolutionary timescales, but rather that the relative proportion of introns belonging to either class grows and shrinks over periods of hundreds of millions of years.

In order to further investigate the evolution of intron lengths, we used a transitive reciprocal best-hit strategy to assemble a dataset of genes we term quartets. Each quartet consists of four genes: a pair of human paralogs and their mouse orthologs. In theory, the orthologous members of each quartet share a more recent common ancestor than do the paralogous members of the quartet. The strong correlation in intron lengths characteristic of orthologous quartet members demonstrates that intron lengths within the vertebrates remain correlated for tens of millions of years following speciation events. Our comparisons of orthologous and paralogous intron lengths in the *Drosophilae* show this to be true of these genomes as well.

### An Intron-Based Molecular Clock

To measure the rate at which intron lengths change, we examined them in the context of the protein clock. Our results show that correlations in the lengths of orthologous introns have declined at a constant rate within the *Drosophilae* during the past 60 million years. We also demonstrate that change in intron length is largely independent of protein evolution. These two results mean that intron lengths provide a molecular measure of time independent of the protein clock. Moreover, we show that the information necessary to employ the intron clock can be extracted from incompletely sequenced genomes. As the distributions in Figure 8 demonstrate, so long as the average contig length exceeds the average intron length, CGL can identify large numbers of orthologous introns even in low-coverage, partially assembled genomes. This result thus also serves to illustrate the ability of CGL to make use of existing annotations in order to extract useful information from unannotated, provisionally assembled genomes—a difficult-to-use but rapidly growing resource for comparative genomics.

The intron and protein clocks complement one another in a number of ways. Rates of change among protein sequences are reasonably constant for any given set of orthologous genes across phyla but vary widely among different gene families. On the other hand, our results show that the speed of the intron clock may vary between phyla, but not between gene families within a genus. These facts mean that the intron clock is well suited for investigating the evolutionary history of gene families. To see why, consider that a collection of genes all having the same intron–exon structures and intron lengths are likely the result of recent duplication events, regardless of whether they encode rapidly or slowly evolving proteins.

### Large-Scale Trends in Intron–Exon Structures

Our analyses of gene structures demonstrate that change in intron–exon structures is subject to greater lineage-specific variation than is protein sequence evolution. The jagged right-hand side of Figure 3B serves to illustrate this fact; by comparison, protein sequence evolution is much more uniform (Figure 3A). Our investigations of intron density distributions (Figure 1C) support a similar conclusion and also provide some indication as to the nature of these rate variations. The shape of the A. gambiae and D. melanogaster intron-density distributions, for example, are suggestive of a long-term trend in the *Diptera* toward intron loss.

Despite the variability in their rate of evolution, the fact that genome-wide trends in intron–exon structures support the same phylogeny as proteome-wide trends in protein sequence similarities (Figure 3) indicates that their power to resolve deep phylogenetic relationships is on par with that of proteins. Moreover, our results show that intron–exon structures evolve largely independently of protein sequences. This is a significant finding, as it means that intron–exon structures offer a source of phylogenetic information independent of protein sequences. Thus, similarities in gene structures can be used to test conclusions drawn from comparative genomic analyses based upon protein sequence similarities. Nevertheless, the greater variability in their rates is cause for caution. Certainly, there is nothing in our data to suggest that phylogenetic trees based upon intron–exon structures should take precedent over protein-based analyses when the two contradict one another.

### Intron Densities

The large numbers of introns and low rate of intron insertion and deletion characteristic of animal genomes make it likely that intron density distributions are among the more slowly evolving traits of any animal genome. Consistent with this hypothesis, the D. melanogaster and A. gambiae distributions are well correlated after 250 million years of independent evolution. Our discovery that intron density distributions (Figure 1C) vary between genomes in a coordinated manner suggests that these distributions provide a new means by which to investigate phylogenetic relationships—one not predicated upon pair-wise comparisons of proteins or gene structures. It also serves to illustrate the power and promise of sequenced genomes and their annotations to cast new light upon the evolution of genes and organisms alike.

## Materials and Methods

### Software.

CGL can be downloaded from http://www.yandell-lab.org/cgl. This site also provides extensive documentation on how to install and use the software. We also employed the Bioperl [52] libraries in our analyses.

### Obtaining the genomes and their annotations.

The human, mouse, and C. elegans genomes were downloaded (August 2004) from the Genomes division of GenBank (ftp://ftp.ncbi.nih.gov/genomes), and converted to Chaos.xml—an input file format to CGL—using the script cx-genbank2chaos.pl provided with CGL. The A. mellifera genome was downloaded from GenBank on 21 July 2005. Th*e D. melanogaster* genome (release 3.1) was obtained from the Berkeley GadFly database [53], converted to Chado-xml (http://www.gmod.org), and then converted to Chaos-xml using the CGL script cx-chadoxml2chaos.pl. The A. gambiae genome was downloaded as an Ensembl database [21] using the CGL script cx-download-enscore.pl and then converted to Chaos.xml using the cx-ensembl2chaos.pl. To convert the *C. intestinalis* genome to Chaos.xml, we obtained its genome and transcript fasta files from the JGI Web site [23], and used sim4 [54] to realign each transcript to the genome, loaded the results into a GadFly database [53], and then converted the resulting annotations to Chaos-xml using the same process that was used for the D. melanogaster genome. The sequences of the five unannotated *Drosophilae* genomes were obtained as follows. The D. simulans W501 assembly (15 March 2004) was downloaded from http://www.dpgp.org; the D. yakuba assembly was downloaded from ftp://genome.wustl.edu/pub/seqmgr/yakuba on 15 March 2004; the D. virilis and D. ananassae assemblies were downloaded from http://rana.lbl.gov/drosophila on 21 June 2004 and 30 June 2004, respectively; the D. pseudoobscura assembly is as used in [29].

Reciprocal best-hit best HSPs were recovered from proteome-versus-proteome BLASTP searches using WU-BLAST [55] cut off: E = 10^−5^; wordmask = seg of the two corresponding nonredundant multi-fasta files. For each search, the database size (WU-BLAST parameter Z) was fixed to the size of the combined nonredundant protein multi-fasta file for all six genomes. Details of the specific analyses are given below.

Figure 1A and 1B summarizes the frequencies of unique intron and unique coding-exon lengths, respectively, for each of the six annotated genomes. When calculating intron density (Figure 1C), in cases where the gene produced more than one transcript but the protein produced by one transcript was a perfect substring of another's, the intron density of the transcript encoding the longer protein was used; if a gene encoded two or more transcripts, both producing two different proteins, the intron density of both was used. For purposes of display, each transcript's intron density was multiplied by 100 and placed in one of 11 bins; these are denoted by the circles on the trend lines in Figure 1C. In Figure 2, the reported BLAST bit score of each reciprocal best-hit best HSP was divided by the length of the shorter aligned sequence (query or subject), and the cumulative distribution function is plotted. For Figures 1 and 2, the resulting data histograms are plotted as curves in order to better illustrate trends, and to facilitate display of data from all six genomes on the same figure. Curves were “smoothed” using Microsoft Excel so that closely overlapping curves did not obscure one another in the figures.

The tree shown in Figure 3A was created using the same sets of reciprocal best-hit best HSPs used to produce Figure 2. The protein alignment portion of each pair-wise set of HSPs was concatenated, and the sum of their bit scores was divided by the length of the resulting concatenated alignment to derive an average number of bits/aligned amino acid pair *(H)* for each pair-wise proteome comparison. Inter-proteome distances were calculated by exponentiation of this value, e.g., D = 2^−*H*^. The resulting 6 × 6 distance matrix was then used to produce a neighbor-joining tree using Phylip [43]. Figure 3B was produced using the same procedure, except that rather than calculate *H,* instead, a related value, *I* = log_2_(P_qs_/(P_q_*P_s_)) was used, where P_qs_ is the observed frequency of aligned splice junctions (to the same aligned amino acid pair), and P_q_ and P_s_ are the frequency of splice junctions in the query and subject portions of the concatenated alignment, respectively. Both trees were bootstrapped by repeating the tree-building process 100 additional times using concatenated alignments of the same length; these were produced by randomly resampling the HSPs with replacement.

To produce Figure 4, all human–mouse, human–*Ciona,* human–*D. melanogaster,* and human–C. intestinalis reciprocal best-hit best HSPs, containing at least one intron in either the query or the subject sequence, were binned by percent amino acid identity (*x*-axis); the average fraction of aligned introns in each bin is denoted by the circle on the trend line. Each of the bins contains at least 100 HSPs from each of the four pair-wise comparisons. To facilitate display of multiple datasets, lines connect data points.

Figure 5 was constructed using the same procedures as Figure 3, but the following procedure was used to obtain a set of concatenated alignments all having the same *H* (1.25 bits/aligned position). First, the reciprocal best-hit best HSPs were sorted by percent identity and the value of *H* was calculated for the HSP collection in toto. Next, an HSP was dropped from the beginning of the sorted list, stored as HSP_a_, and *H* was recalculated and stored as (*H*_1_). HSP_a_ was returned to the list, and an HSP was dropped from the end of the sorted list, stored as HSP_b_, and *H* was recalculated and stored as (*H*_2_). Depending upon which value (*H*_1_ or *H*_2_) was closest to the target value of 1.25 bits, either HSP_a_ or HSP_b_ was permanently dropped from the list, and the process was repeated until the value of *H* for the remaining HSPs converged upon the target value ± 0.005 bits. This value was then used to produce the tree shown in Figure 5A. *I* was then calculated using this set of HSPs to produce the tree shown in Figure 5B.

To extract orthologous introns from unannotated genomes, each annotated D. melanogaster protein was searched against a genome assembly using WU-TBLASTN [55] (cut off: E = 1e^−5^; wordmask = seg). For all searches, the database size (Z) was set to 128,000,000 nt, the approximate size of the D. melanogaster euchromatic genome. CGL was then used to infer whether or not the details of the resulting TBLASTN HSPs of the best hit to the target genome were consistent with the presence of an intron in the target genome at the same position as an annotated splice junction on the *melanogaster* protein. Orthologous introns were counted as found only if the portion of the TBLASTN alignment flanking each inferred intron junction had greater than 25% identity and was at least 15 amino acids long—and only then if the putative intron began with the sequence GT and ended with an AG dimer; the procedure was thus quite stringent. The length distributions of these introns are shown in Figure S1. Current D. melanogaster annotation standards forbid the creation of an annotation having an intron less than 40 bases in length [19]. We adopted the same rule when constructing Figure 8, and thus excluded every case in which the inferred intron was less than 40 nt in length. It is worth noting that we did identify some introns smaller than 40 bp in the unannotated genomes; however, upon closer inspection, most but not all appeared to be due to either assembly artifacts or pseudo-genes. To facilitate display of multiple datasets, the resulting histograms are plotted as lines. Figure 7A and the panels in Figure 8 were produced by plotting the length of the annotated D. melanogaster intron on the *x*-axis and the length of its inferred ortholog on the *y*-axis. Introns containing repeats (Figure 7A) were identified using RepeatMasker [47] together with the BDGP transposon library [56].

## Supporting Information

Figure S1 Annotated and Inferred Intron Lengths for Six *Drosophila* Species All intron lengths are inferred, with the exception of D. melanogaster.
dana, *D. ananassae;* dmel, *D. melanogaster;* dpse, *D. pseudoobscura;* dsim6, D. simulans (strain 6); dvir, *D. virilis;* dyak, *D. yakuba. x*-axis, intron length (log _10_); *y*-axis, frequency.
(2.1 MB PSD)

Figure S2 Neighbor-Joining Tree of Pair-Wise Correlations in Orthologous Intron Lengths Pair-wise Spearman correlation coefficients were used as a similarity measure. Bootstraps were produced by randomly resampling intron pairs with replacement. All intron lengths are inferred, with the exception of D. melanogaster. The long D. simulans branch length is a consequence of the low sequence coverage and the provisional nature of its genomic assembly.
dana, *D. ananassae;* dmel, *D. melanogaster;* dpse, *D. pseudoobscura;* dsim6, D. simulans (strain 6); dvir, *D. virilis;* dyak, *D. yakuba.*
(39 KB PSD)

Figure S3 Similarity in Orthologous Intron Lengths Is Little Influenced by the Intensity of Selection on Flanking Exons *x*-axis, average D. melanogaster–D. yakuba K_a_/K_s_ for each pair of exons flanking each orthologous intron pair. *y*-axis, fractional difference in length of the corresponding orthologous D. melanogaster–D. yakuba intron pair, L_c_; where L_c_ = [(L_i_ + L_j_) − |L_i_ – L_j_|] / (L_i_ + L_j_); and L_i_ and L_j_ refer to length of orthologous introns i, and j, respectively. If the two introns are the same length, L_c_ equals 1. If one member of the pair is twice the length of the other, L_c_ equals 0.5. Thus L_c_ provides a simple means to associate a similarity value with each pair of orthologous introns. For purposes of display 1 − L_c_ is plotted so that two introns having exactly the same length, flanked by exons with a K_a_/K_s_ = 0 will lie at the graph's origin.
Orange line, best-fitting linear regression (y = 0.0457x + 0.0863; R^2^ = 0.0015). No significant Spearman correlation coefficient was observed for these data.
(2.6 MB PSD)

## Abbreviations

HSP: high-scoring segment pair
LOD: log odds ratio
nt: nucleotides
